# Betrixaban is a broad anti-virus inhibitor by activating innate immunity

**DOI:** 10.3389/fcimb.2025.1603530

**Published:** 2025-08-21

**Authors:** Shiyu Hu, Yang Zhao, Xingyu Chen, Haocheng Wang, Wenjun Hu, Rong Huang, Jian Yang, Chenxi Niu, Xuefei Guo, Fuping You

**Affiliations:** ^1^ Institute of Systems Biomedicine, Department of Immunology, School of Basic Medical Sciences, Beijing Key Laboratory of Tumor Systems Biology, National Health Commission (NHC) Key Laboratory of Medical Immunology, Peking University Health Science Center, Beijing, China; ^2^ Department of Orthopedics and Traumatology, Nanchong Hospital of Traditional Chinese Medicine, Nanchong, China; ^3^ Institute of Basic Medicine and Forensic Medicine, North Sichuan Medical College, Nanchong, China; ^4^ Department of Pathology, Xiangya Hospital, School of Basic Medical Sciences, Central South University, Changsha, China

**Keywords:** broad-spectrum antiviral, Betrixaban, FXa, innate immunity, VSV

## Abstract

The innate immune system serves as the first line of defense against viral infections. Type I interferon (IFN-I) signaling, in particular, plays a crucial role in mediating antiviral immunity. Here, we identify Betrixaban (BT), a novel small-molecule compound that activates innate immune responses, leading to broad-spectrum antiviral effects. BT induces IFN-β production and upregulates interferon-stimulated genes (ISGs), effectively suppressing the replication of multiple viruses, including vesicular stomatitis virus (VSV), herpes simplex virus type 1 (HSV-1), murine hepatitis virus strain A59 (MHV-A59), encephalomyocarditis virus (EMCV), and influenza A virus (IAV). BT’s antiviral activity relies on innate immune activation, with IRF3 playing a key role. The antiviral effect was significantly reduced upon loss of ISGs induction, including Mx1 and Mx2. *In vivo*, BT treatment markedly induced IFNB1 expression across multiple mouse tissues and significantly inhibited viral replication in VSV-infected wild-type mice, confirming the essential role of innate antiviral immune activation. These findings establish BT as a potent stimulator of the innate immune system, demonstrating broad-spectrum antiviral potential and highlighting its promise as a therapeutic agent.

## Graphical Abstract

Cartoon schematic of the BT-regulated antiviral immunity pathway. BT triggers a TBK1/IRF3-dependent immune response, thereby inhibiting viral replication both *in vivo* and vitro.

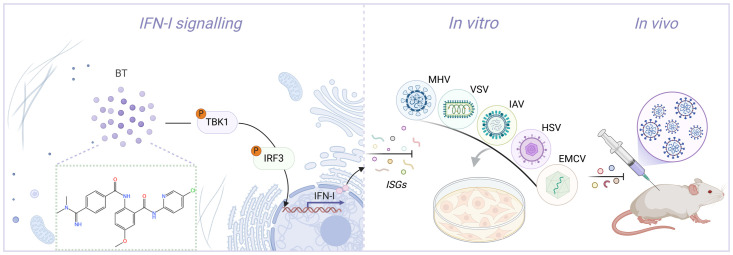

## Introduction

The COVID-19 pandemic, caused by severe acute respiratory syndrome coronavirus 2 (SARS-CoV-2), has presented unprecedented global health challenges and necessitated urgent advancements in antiviral therapeutics (Emrani et al., 2021; V’kovski et al., 2021). Traditional approaches to antiviral drug development are often lengthy and complex, making the repurposing of existing medications for new applications an attractive strategy. Viral infection is a multifaceted process. After binding to and entering host cells, the virus hijacks host proteases and other cellular proteins to replicate and spread (Hoenen and Groseth, 2022). This process triggers the host cell’s innate immune system, leading to subsequent antiviral immune responses (Loo and Gale, 2007). These cell-autonomous antiviral mechanisms aim to curtail viral propagation by arresting the cell cycle, blocking host cell mRNA translation, dismantling infected cells, and limiting the resources available for viral replication (Stetson and Medzhitov, 2006; Pichlmair and Sousa, 2007; Channappanavar, 2022). Therefore, targeting host factors, particularly those involved in immune modulation, may represent a prominent strategy to prevent viral infection (Maarifi et al., 2022).

The innate immune system plays an indispensable role in the early detection and response to viral infections (Pichlmair and Sousa, 2007). Pattern recognition receptors (PRRs), such as Toll-like receptors (TLRs), retinoic acid-inducible gene-I (RIG-I)-like receptors (RLRs), and cyclic GMP-AMP synthase (cGAS), recognize viral components and initiate signaling cascades that lead to the production of type I interferon (IFN-I) and other cytokines, which are essential for establishing antiviral states in infected tissues (Sadler and Williams, 2008; Zevini et al., 2017; Ablasser and Hur, 2020; Li et al., 2021). IFN-I, particularly IFN-α and IFN-β, plays a central role in antiviral defense by inducing the expression of interferon-stimulated genes (ISGs), which restrict viral replication and enhance immune responses (Dalskov et al., 2023). This signaling cascade is mediated mainly by the activation of TBK1 and IRF3, leading to the transcription of antiviral effectors (Lin and Young, 2014; Milora and Rall, 2019). In addition to its antiviral role, IFN-I modulates immune responses in various biological contexts, including inflammation, immune evasion, and tumor immunity (Gajewski and Corrales, 2015; Lazear et al., 2019; Yu et al., 2022). Therefore, small-molecule compounds capable of stimulating IFN-I production and ISGs induction hold significant potential as broad-spectrum antiviral agents.

Betrixaban (BT), a highly selective direct oral inhibitor of Factor Xa (FXa), is an o-aminobenzamide structural compound developed by Merck for the prevention of venous thromboembolism and related complications in acutely ill patients (Patel et al., 2016). More than 80% of BT is excreted in its original form in the bile, making it the least renally excreted among the xaban anticoagulant drugs, including Rivaroxaban, Apixaban, and Edoxaban. Based on this metabolic property, BT is of great research interest in cardio-cerebrovascular patients with renal insufficiency or severe impairment and plays a vital role in anticoagulation (Zhang et al., 2009; de Vries et al., 2020). In addition to its clinical applications, BT exhibits extensive pharmacological effects, including antiviral and anti-inflammatory activities (Al-Horani, 2020). Recent studies have identified BT as a potential inhibitor of coronavirus main protease (Mpro) capable of significantly reducing virus-induced cytopathic effects by virtual screening technology and *in vitro* drug activity assay experiments (Ambrosio et al., 2023). A large-scale, high-throughput computational screen in the Selleckchem FDA-approved drug library revealed that BT has the ability to bind to TMPRSS2, a cellular receptor for most coronaviruses. Inhibition of TMPRSS2 protein activity blocks viral invasion (Kim et al., 2020). Furthermore, BT was identified as a repurposed drug candidate against SARS-CoV-2 by using artificial intelligence and machine learning (Rajput et al., 2021). This finding suggests BT’s potential as a coronavirus therapeutic agent. However, the broad-spectrum antiviral effects and mechanisms of BT remain poorly studied.

Here, using a firefly luciferase (Luc) reporter system, a library of small molecules from MCE was screened to identify IFN-I activators. We found that the compound BT effectively activated innate immunity. Subsequently, we demonstrated for the first time that BT remarkably suppressed a wide range of viral replications *in vitro*, including vesicular stomatitis virus (VSV), encephalomyocarditis virus (EMCV), MHV strain A59 (MHV-A59), influenza A virus (IAV), and herpes simplex virus type 1 (HSV-1). Further analysis revealed that this inhibition occurred through type I interferon signaling and was independent of FXa. Mechanistically, BT enhanced IFN-I responses via the TBK1-IRF3 signaling pathway, inducing robust ISGs expression and antiviral immunity. Additionally, in VSV-infected mice, BT significantly inhibited the replication of VSV and improved survival, confirming its potential as a systemic antiviral agent. Collectively, our findings indicate that BT is a novel innate immune activator and a broad-spectrum antiviral inhibitor, positioning it as a promising drug candidate for viral infectious diseases that may emerge in the future.

## Materials and methods

### Antibodies and reagents

Rabbit anti-VSV-G-Tag antibodies were purchased from Boster (catalog no. BM3890). Rabbit antibodies against TBK1 (catalog no. 28397-1-AP) and Beta Tubulin (catalog no. 10094-1-AP), along with mouse antibodies targeting Beta Actin (catalog no. 66009-1-Ig) and the mCherry tag (catalog no. 68088-1-Ig), were procured from Proteintech. Additional antibodies, including rabbit phospho-TBK1/NAK (Ser172) (D52C2) (catalog no. 5483), phospho-IRF-3 (Ser396) (D6O1M) (catalog no. 29047), and IRF-3 (D6I4C) (catalog no. 11904), were obtained from CST. The secondary antibody consisted of HRP-conjugated Affinipure Goat Anti-Rabbit IgG (H+L) (catalog no. SA00001-2) and HRP-conjugated Affinipure Goat Anti-Mouse IgG (H+L) (catalog no. SA00001-1), both procured from Proteintech. ABclonal supplied active recombinant mouse CSF-2/GM-CSF protein (catalog no. RP01206). Human recombinant IFN-β (catalog no. HY-P73128) was purchased from MCE.

### Cell culture

The HeLa, Vero, A549, J774A.1, RAW264.7, HT29, and HEK293T cell lines were sourced from the American Type Culture Collection (ATCC). Bone marrow-derived macrophages (BMDMs) were obtained by isolating cells from the femurs and tibiae of 8-week-old mice, followed by culturing them in the presence of GM-CSF for 7 days to induce macrophage differentiation. The 2fTGH-interferon-stimulated response element (ISRE) cell line, which stably expresses an ISRE-driven luciferase reporter, was established by transfecting 2fTGH cells with the ISRE-luciferase plasmid, followed by stabilization. Vero, A549, J774A.1, RAW264.7, HEK293T, and BMDMs were maintained in Dulbecco’s Modified Eagle’s Medium (DMEM; EallBio, 03.1002C), while HeLa and HT29 cells were cultured in RPMI 1640 medium (EallBio, 03.4001C). All culture media were supplemented with 10% fetal bovine serum (FBS, TransGen Biotech, FS201-0) and 1% penicillin-streptomycin (TransGen Biotech, FG101-01). Cells were incubated at 37°C in a humidified incubator with 5% CO2. Cell lines utilized were maintained at low passage numbers (below 15 passages) to ensure consistent cellular characteristics and reproducibility.

### Virus infection and propagation

Cells at 70-80% confluence were infected with the following viruses at the indicated multiplicity of infection (MOI): Vesicular Stomatitis Virus (VSV, MOI = 0.1), Herpes Simplex Virus 1 (HSV-1, F strain, MOI = 0.5), Encephalomyocarditis Virus (EMCV, MOI = 0.1), Mouse Hepatitis Virus (MHV, A59 strain, MOI = 0.1), Influenza A Virus (IAV, PR8 strain, MOI = 0.1), and VSV expressing green fluorescent protein (VSV-GFP, MOI = 0.1).

Virus stocks were prepared as follows: VSV Indiana strain and VSV-GFP, generously provided by Prof. J. Rose (Yale University), were propagated in Vero cells. HSV-1 strain 17, kindly provided by Prof. Zhengfan Jiang (Peking University), was cultured in the same cell line for viral amplification. MHV-A59 strain (ATCC VR-764) and EMCV (ATCC VR-129B) were purchased from ATCC and propagated in 17CL-1 and Vero cells, respectively. IAV strain PR8, kindly provided by Prof. Qiang Feng (Fudan University), was propagated in fertile chicken eggs under standard conditions. Briefly, for VSV, HSV-1, EMCV, and MHV-A59, cells were infected with each virus and incubated at 37°C until extensive cytopathic effect (CPE) was observed (typically within 24-48 h). After extensive CPE was observed, culture supernatants and infected cells underwent three freeze-thaw cycles (−80°C/37°C) to facilitate virus release. The resulting lysates were centrifuged at 3,000 × g for 15 minutes at 4°C to remove cellular debris, aliquoted, and stored at −80°C. In contrast, for IAV, fertile chicken eggs were inoculated via the allantoic cavity with virus suspension. Eggs were incubated at 37°C (55-65% humidity) for 48-72 h. Subsequently, eggs were chilled at 4°C overnight, and allantoic fluid was aseptically harvested. Virus-containing allantoic fluid was clarified by centrifugation at 3,000 × g for 10 minutes at 4°C, aliquoted, and stored at −80°C. Virus titers for all strains were determined by standard plaque assay or hemagglutination assay prior to experiments.

### Mice and *in vivo* virus infection

All animal care and procedures were conducted under the Guide for the Care and Use of Laboratory Animals by the Chinese Association for Laboratory Animal Science. The Animal Care Committee of Peking University Health Science Center approved the protocols (permit number: LA 2016240).

Wild-type (WT) C57BL/6J mice were obtained from the Department of Laboratory Animal Science, Peking University Health Science Center. *Irf3* knockout mice were generously provided by Prof. Zhengfan Jiang (Peking University). All animals were maintained with unrestricted access to clean water and nutritious feed.

Age- and sex-matched C57BL/6J littermates were used for all *in vivo* experiments. Eight-week-old mice were infected intraperitoneally (i.p.) with VSV at a dose of 1 × 10^8^ plaque-forming units (PFU) per mouse. Following infection, mice received daily intraperitoneal injections of Betrixaban (BT, 10 mg/kg/day, n = 8 per group) or phosphate-buffered saline (PBS) as a vehicle control for three consecutive days. At 48 h post-infection, blood samples were collected to measure VSV nucleocapsid protein (VSV-N) mRNA levels via quantitative reverse transcription PCR (RT-qPCR). Body weight and survival rates were monitored daily. On day 4 post-infection, all mice were euthanized using CO2 inhalation for subsequent analyses. To evaluate the induction of type I interferon (IFN-I) responses by BT *in vivo*, an additional set of C57BL/6J mice received a single i.p. injection of BT (10 mg/kg, n = 3 per group) or PBS control, and tissues were collected 24 h later for IFN-I-related gene expression analysis by RT-qPCR.

### Construction of FXa expression plasmid

Human FXa cDNA was cloned into the pcDNA3.1-mCherry expression vector using a seamless cloning and assembly kit (Transgen, CU101-01), following the manufacturer’s standard protocol. The coding sequences were verified in their entirety through Sanger sequencing to ensure the accuracy and integrity of the cloned plasmid. The expression of fusion protein was detected by Western blotting and fluorescence assay.

### Luciferase assay

A customized library of approximately 400 small molecules related to carbohydrate metabolism was purchased from Selleck (USA). All compounds were stored as 10 mM stock solutions in dimethyl sulfoxide (DMSO) or water at −80°C until use. For screening assays, cells seeded in 12-well plates were treated with Sendai virus (SeV, MOI = 0.1) or individual library compounds (60 μM) for 12 h at 37°C. After treatment, cells were lysed directly in the wells using a lysis buffer, per the manufacturer’s instructions (Transgen, FR203-01). The lysates were collected, and luciferase activity was quantified using a GloMax^®^ 20/20 Luminometer (Promega). Relative luciferase activity was expressed as fold change relative to the control (set as 1.0).

### RNA isolation and RT-qPCR

TRIzol reagent (TIANGEN, A0123A01) was used to isolate total RNA from cells or tissues with various treatments or infections. The extracted RNA was subsequently reverse transcribed into complementary cDNA with HiScript II RT SuperMix (Vazyme, R223-01). Target gene expression levels were measured through RT-qPCR analysis using SYBR Green qMix (Vazyme, Q311). The housekeeping gene β-actin (ACTB) was used to normalize the data. Fold changes in gene expression were calculated using the 2^-ΔΔCT method. The control group was set as 1 to serve as the baseline for relative expression. This calculated fold change reflects the relative expression level of the target gene in the test samples compared to the control samples. Detailed primer sequences used in this study are listed in **Supplementary Table S1**.

### Western blotting

Cells were lysed with RIPA Buffer (MCE, HY-K1001) containing EDTA-free Protease Inhibitor Cocktail and Phosphatase Inhibitor Cocktails I and II. The cell lysates (10 to 30 μg) were separated by SDS-polyacrylamide gels and subsequently transferred to nitrocellulose membranes (Beyotime, FFN08). Following blocking, the membranes were immunoblotted with the indicated primary antibodies (1:1000), subsequently with HRP-conjugated secondary antibodies (1:10000). The proteins were detected using an enhanced ECL system (EallBio, 07.10009-50).

### Cell cytotoxicity assay

Seed target cells into a 96-well plate at a density of 2×10³ cells per well in 100 μL of complete medium. Treat the cells with increasing concentrations of BT (MCE, HY-10268) (0, 3.125, 6.25, 12.5, 25, 50, 100, 150, and 200 μM) and incubate at 37°C in a humidified 5% CO_2_ incubator for 12, 24, 36, or 48 h. Subsequently, add 10 μL of CCK-8 reagent (YEASEN, 40203ES60) to each well and gently shake the plate to mix. After incubation at 37°C for 1 h, measure the absorbance at 450 nm using a microplate reader. Untreated cells cultured in parallel served as negative controls (defined as 100% viability). Blank wells containing medium without cells were included for background correction. Relative cell viability (%) was calculated according to the following formula: OD [(experimental -blank)/(control -blank)] × 100%.

### Fluorescence assay

Cells were either treated with small molecules (0-100 μM, 0-24 h) or transfected with plasmids (1 μg, 48 h). After infection with VSV-GFP at an MOI of 0.1 for 12 h, fluorescence signals were acquired under a fluorescence microscope (CKX53, Olympus) with an excitation wavelength of 488 nm or 530 nm. Images were acquired at a magnification of 100×.

### Flow cytometry analysis

Following viral infection, cells were washed twice with PBS and trypsinized to obtain a single-cell suspension. After centrifugation at 1600 rpm for 5 minutes, the cell pellets were resuspended in flow cytometry stain buffer for further analysis. Infection efficiency was determined by quantifying GFP-positive cells using a flow cytometer (BD Biosciences). Uninfected cells were analyzed in parallel to define GFP-negative populations.

### siRNA transfection and FXa gene silencing

Small interfering RNAs (siRNAs) targeting FXa and a non-targeting control siRNA were procured from Hippobio company. 2fTGH cells were transiently transfected with siRNAs (100 nM) using FuGENE^®^ SI Transfection Reagent (Promega, E9311) according to the manufacturer’s instructions. After 12 h of incubation, the supernatant was replaced with fresh DMEM, and the cells were cultured for an additional 48 h. Gene silencing efficiency was assessed by RT-qPCR.

### CRISPR-Cas9 system

The IRF3 knockout 2fTGH cell line was generated using the CRISPR-Cas9 system. High-efficiency and high-specificity guide RNAs (gRNAs) were designed and selected using an online CRISPR design tool (https://www.genscript.com/tools/gRNA-design-tool). The oligos were annealed and cloned into the lentiCRISPR v2 vector, which had been digested with the BsmBI enzyme (NEB).

For lentivirus production, 293T cells were cotransfected with the following plasmids: lentiCRISPR v2 (2400 ng), packaging plasmid psPAX2 (800 ng, Addgene 12260), envelope plasmid VSV-G (800 ng, Addgene 8454), and PEI (1600 ng). The transfection mixture was incubated at 37°C for 72 h. Viral supernatants were then collected and used to infect 2fTGH cells. 48 h post-infection, the cells were refreshed with fresh culture medium and selected using 3 µg/mL puromycin. Successful knockout of IRF3 was validated by Sanger sequencing and Western blotting. Primer sequences used in this study are listed in **Supplementary Table S1**.

### Virus inhibition assay

Cells were seeded into 24-well plates and infected with the virus at an MOI of 0.1. Serial dilutions of BT (0-100 μM), IFN-β (1000 U/mL), Betrixaban maleate (MCE, HY-10268A, 50 μM), or Rivaroxaban (MCE, HY-50903, 50 μM) were added to the infected cells. The plates were incubated at 37°C for 12 or 24 h. After incubation, supernatants containing virions were collected for plaque assay. The infected cells were washed twice with PBS and harvested for subsequent analysis by RT-qPCR or Western blotting.

### Plaque assay

Confluent monolayers of Vero cells were plated in 24-well plates and infected with 10-fold serial dilutions of virus for 1 h at 37°C to allow adsorption. After removing the inoculum, cells were washed twice with PBS and overlaid with DMEM containing 0.5% carboxymethyl cellulose (CMC) (Sigma, 419338). Plates were incubated at 37°C for 48 h for plaque formation. The cells were then fixed with 4% paraformaldehyde and stained with 1% crystal violet to visualize plaques. Viral titer was calculated as PFU/mL based on the plaque counts, dilution factor, and inoculum volume.

### RNA-seq and data analysis

Total RNA was extracted using the high-throughput RNA extraction kit (TIANGEN, A0123A01). Quality control, library preparation, sequencing, and data analysis were performed by Suzhou GENEWIZ Biotechnology company (https://www.genewiz.com.cn/), following established standard protocols. The gene count matrix was generated using featureCounts (v2.0.0). The count data was normalized using the Fragments Per Kilobase Million (FPKM) formula. Differential expression analysis was conducted using the DESeq2 R package (v1.38.3), with the thresholds set at |log2(FoldChange)| > 2 and p-value < 0.05 to identify significantly differentially expressed genes (DEGs).

### GO annotation and KEGG pathway enrichment analysis

Gene ontology (GO) annotation and Kyoto Encyclopedia of Genes and Genomes (KEGG) pathway analysis were used to analyze the enrichment degree of DEGs. The analysis was performed with the clusterProfiler R package, using the enrichGO and enrichKEGG functions. Additionally, the DAVID database (v6.8, https://david.ncifcrf.gov/summary.jsp) was used as an alternative tool. Terms with a false discovery rate (FDR) below 0.01 were considered significantly enriched.

### Gene set enrichment analysis

Gene Set Enrichment Analysis (GSEA) is a computational method used to identify whether predefined gene sets exhibit significant and coordinated differences between two biological states. Unlike traditional approaches that focus only on DEGs, GSEA analyzes all genes, regardless of their significance levels. In this study, GSEA was conducted using the clusterProfiler R package. The gseaplot2 function from the same package was used to visualize the enrichment results.

### Statistical analysis

All the bar graphs were generated by Prism 6.0 software (GraphPad Software, La Jolla, CA). Statistical analyses were analyzed with t test and One-way ANOVA, and the results are presented as the mean ± SEM. Survival comparisons were performed using the log-rank (Mantel-Cox) test. All experiments were independently repeated at least three times. The p values < 0.05 were considered statistically significant (*), p values < 0.01, and p values < 0.001 were regarded as highly statistically significant (** and ***).

## Results

### Betrixaban drives innate immune response and ISGs expression

**Figure 1:**
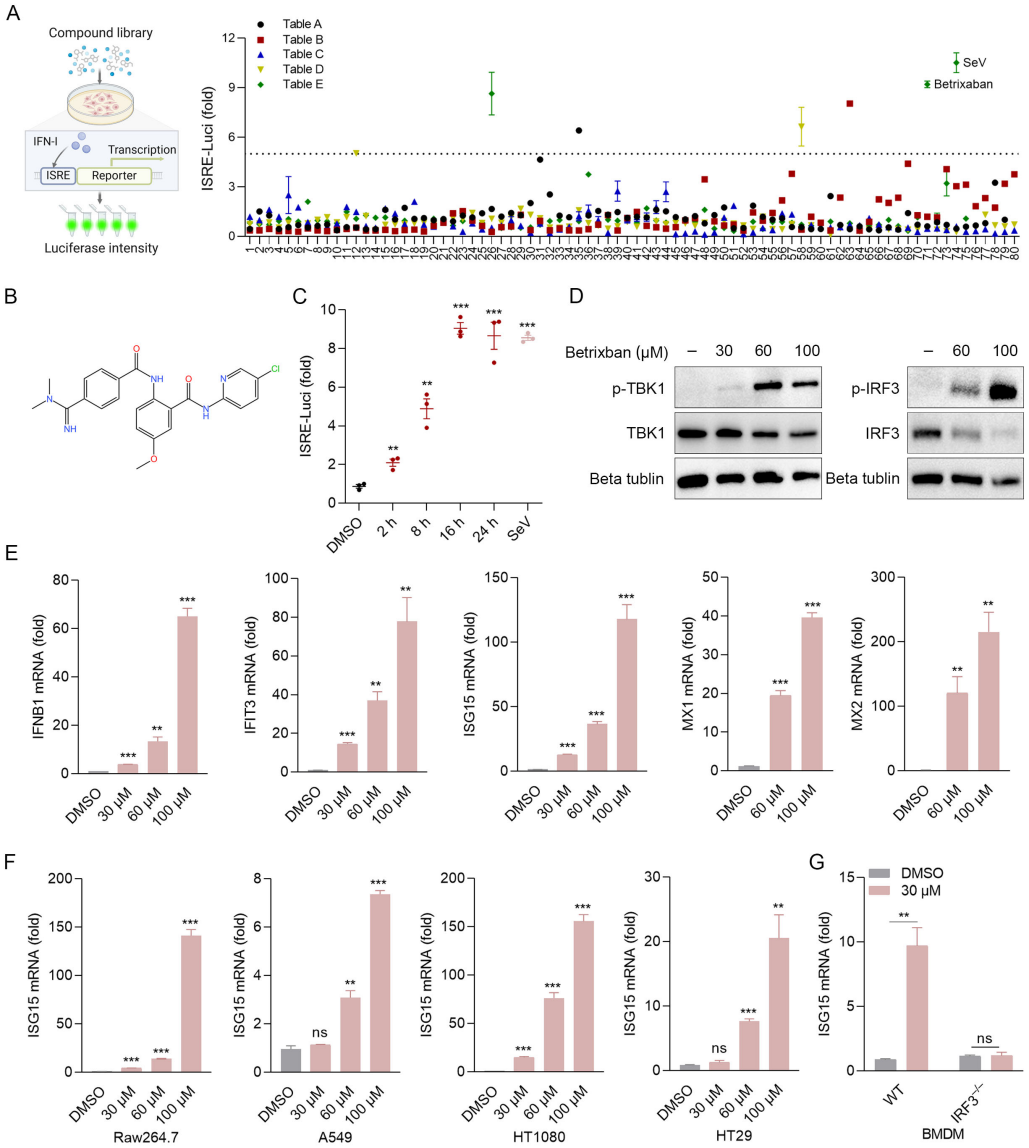
Betrixaban (BT) activates the innate immune response. **(A)** Schematic illustration of the drug-of-addiction assay. IFN-β expression was measured by luciferase expression in 2fTGH-ISRE cells following a 12-h exposure to media containing 60 μM compounds from the small molecule drug library. Relative luciferase activity was expressed as fold change relative to the control (DMSO, set as 1.0). Compound E71* (Betrixaban, BT) exhibited the highest efficacy and was selected for further characterization. **(B)** The Chemical structure of BT. **(C)** Expression of IFN-β-dependent luciferase in 2fTGH cells treated with BT (60 μM) for 0-24 h. Data expressed as fold change vs. DMSO control. **(D, E)** Western blotting and RT-qPCR analysis of type I interferon (IFN-I) signaling activity in 2fTGH cells treated with increasing doses of BT (30, 60, or 100 μM) for 12 h. **(F)** RT-qPCR analysis of ISG15 mRNA expression levels in multiple cell types treated with BT (30, 60, or 100 μM for 12 h). Cell lines: RAW264.7, A549, HT 1080, and HT29. **(G)** Isg15 mRNA levels in wild-type (WT) or *Irf*3 knockout (Irf3^–/–^) murine bone marrow-derived macrophages (BMDMs) post-BT treatment (30 μM, 12 h). Data normalized to ACTB and expressed as fold change relative to DMSO-treated control (set as 1). Data are presented as mean ± SEM, with n = 3. **P < 0.01; ***P < 0.001. ns, not significant.

To identify potential cell-permeable activators of the innate immune response, a metabolism-focused small molecule library was screened to detect the production of IFN-β in the 2fTGH-ISRE-Luciferase cell line (**Figure 1A**). We identified a small number of molecules, including BT (**Figures 1A, B**), that induced IFN-β production in 2fTGH cells following 12 h of co-incubation. Notably, the levels of IFN-β induced by BT alone were comparable to those caused by SeV particles (**Figure 1A**). Therefore, we selected BT for subsequent analyses. A luciferase reporter assay showed that BT treatment triggered IFN-β promoter activity in a time- and dose-dependent manner within a non-cytotoxic dosage range (**Figure 1C**; **Supplementary Figures S1A–S1E**). Additionally, treatment of 2fTGH cells with BT induced the phosphorylation of both TBK1 and IRF3, as well as the expression of their downstream ISGs, indicating a strong activation of the innate immune signaling pathway (**Figures 1D, E**). Interestingly, BT treatment resulted in a dose-dependent suppression of TBK1 and IRF3 protein expression levels, which may imply a potential negative feedback mechanism to prevent excessive immune activation or limit prolonged inflammatory response, consistent with prior studies on similar pathways (Higgs and Jefferies, 2008; Cui et al., 2012; Hu et al., 2023) (**Figure 1D**). Moreover, BT also induced ISG15 expression in variable cell types, including RAW264.7, HT29, A549, and HT1080 cells (**Figure 1F**). Likewise, RT-qPCR analysis demonstrated that BT markedly activated the type I interferon (IFN-I) response in mouse macrophages, but did not exhibit such activity in IRF3-deficient (Irf3^–/–^) murine bone marrow-derived macrophages (BMDMs) (**Figure 1G**). The results show a significant dose-dependent elevation in the mRNA levels of IFNB1 and ISGs in various cells after treatment with BT, demonstrating that BT is a potent activator of the innate immune system.

### BT suppresses the replication of VSV *in vitro*

**Figure 2:**
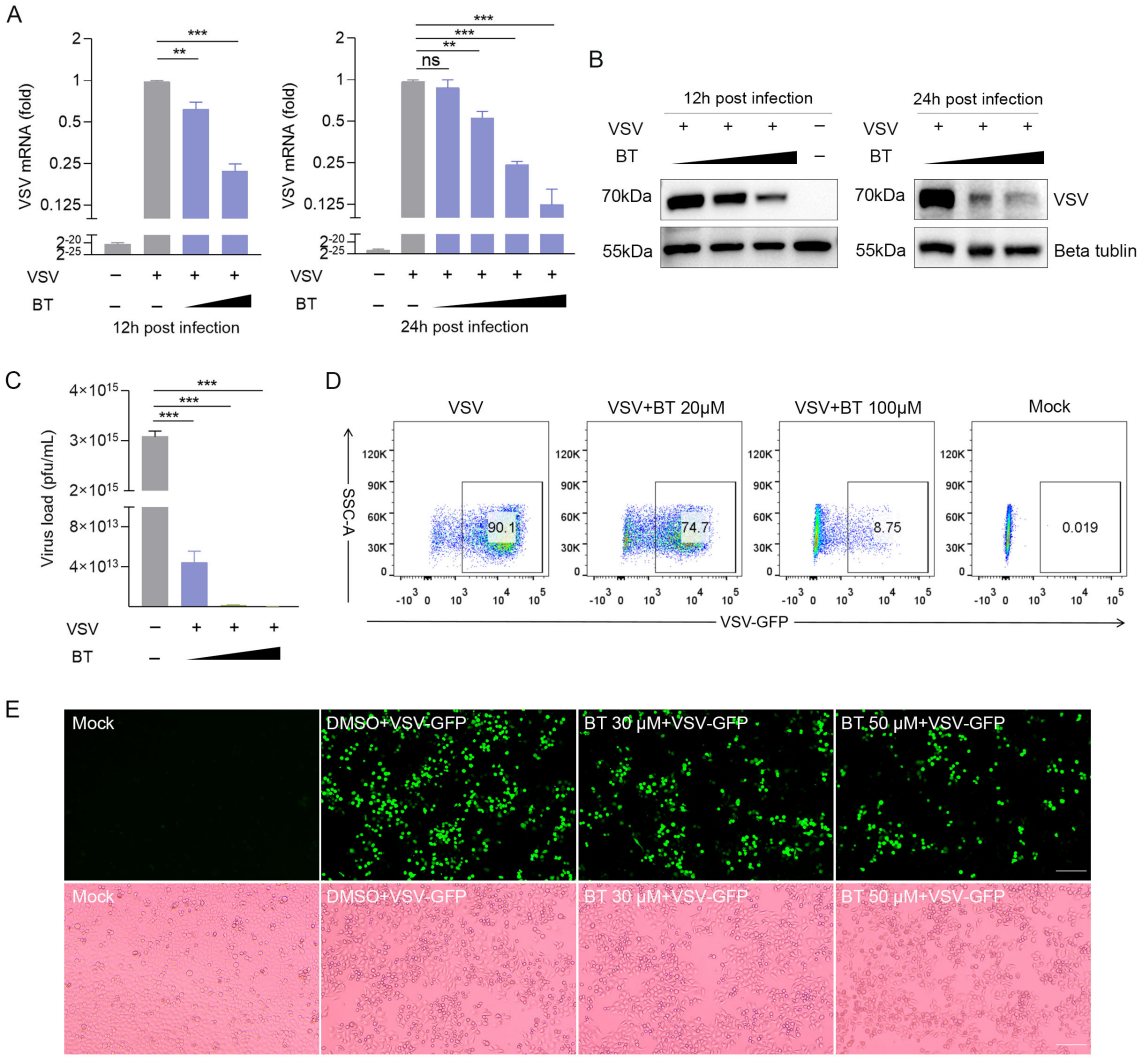
BT restricts the VSV infection in 2fTGH cells. **(A)** RT-qPCR analysis of VSV-N mRNA levels in 2fTGH cells infected with VSV (MOI = 0.1) and treated with DMSO or increasing concentrations of BT (30 and 60 μM for 12 h; 10, 30, 60, and 100 μM for 24 h). Black triangles indicate increasing BT concentrations. Data normalized to ACTB. Fold changes relative to VSV-treated control samples were calculated using the 2^-ΔΔCT method. **(B)** Western blotting analysis of VSV glycoprotein (G) protein expression in 2fTGH cells infected with VSV (MOI = 0.1) and treated with DMSO or BT (30 and 60 μM for 12 h; 60 and 100 μM for 24 h). **(C)** Plaque assay quantification of infectious viral particles from supernatants of BT-treated cells for 12 h. Culture supernatants were serially diluted and adsorbed onto fresh 2fTGH cells for 1 h, then overlaid with 0.5% carboxymethyl cellulose and incubated for an additional 48 h. **(D)** HeLa cells infected with VSV-GFP at an MOI of 0.1 and treated with BT (20 and 100 μM) for 24 (h) The percentage of VSV-GFP-positive cells was determined by flow cytometry analysis. **(E)** Representative bright-field and green fluorescence images of HeLa cells infected with VSV-GFP (MOI = 0.1, 12 h) with BT (0-60 μM). Scale bar, 100 μm. Data are presented as mean ± SEM, with n = 3. **P < 0.01; ***P < 0.001. ns, not significant.

Innate immune responses, particularly those mediated by IFNs, play a considerable role in host defense against viral infections. As BT exhibited a notable capacity to activate IFNs, we subsequently explored the antiviral properties of BT *in vitro*. To assess the inhibitory effects of BT on VSV, 2fTGH cells were infected with VSV and treated with different concentrations of BT. RT-qPCR and Western blotting results demonstrated that BT effectively suppressed VSV replication at concentrations of 60 µM and above at both 12 and 24 h post-infection (**Figures 2A, B**). Consistent with these findings, microscopic examination revealed marked attenuation of virus-induced cytopathic effects (CPE) following BT treatment. Specifically, infection-associated morphological changes, including cell enlargement, rounding, detachment from the culture surface, and subsequent cellular fragmentation, were significantly alleviated after BT administration (**Supplementary Figure S2A**). In addition, plaque assay results showed that BT significantly reduced the number of VSV plaques as the concentration of the drug increased in Vero cells (**Figure 2C**; **Supplementary Figure S2B**). Flow cytometry analysis of GFP-positive cells in VSV-GFP-infected HeLa cells revealed that BT treatment resulted in noticeable inhibition of VSV infection (**Figure 2D**). Similar results were confirmed by fluorescence images (**Figure 2E**). Importantly, BT exhibited antiviral potency comparable to that of IFN-β, a cytokine clinically employed in the treatment of various viral infections by inducing ISGs, suggesting that BT may exert its antiviral effects via similar IFN-dependent pathways (**Supplementary Figures S2C, S2D**). Overall, these data demonstrate that BT effectively restricts VSV infection in a dose-dependent manner.

### BT exhibits a pan-viral inhibitory activity *in vitro*

**Figure 3:**
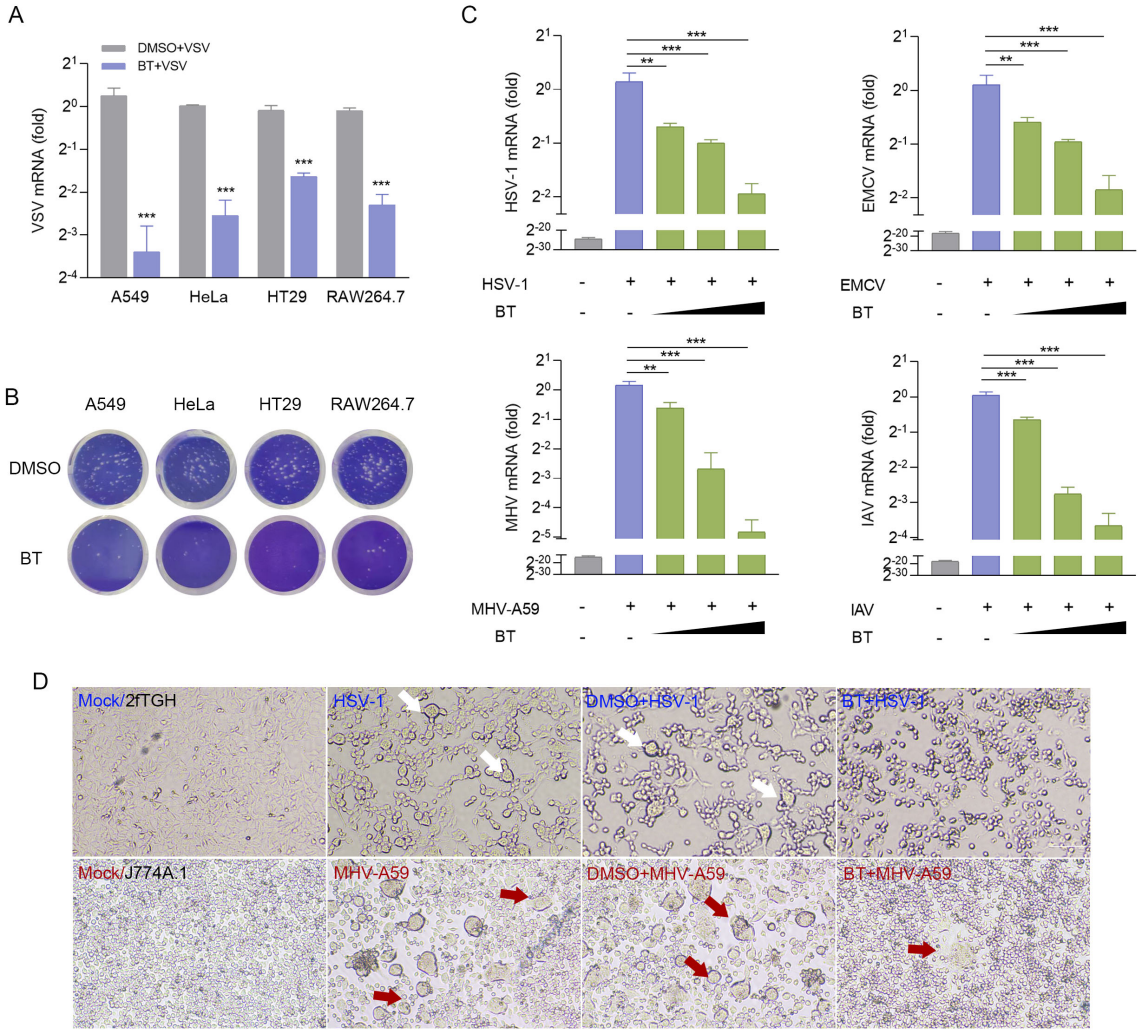
BT inhibits a range of viral infections *in vitro*. **(A)** RT-qPCR analysis of VSV-N mRNA levels in VSV-infected (MOI = 0.1) A549, HeLa, HT29, and RAW264.7 cells treated with BT (50 μM) or DMSO (vehicle control) for 12 h. Data normalized to ACTB and expressed as fold change relative to control (VSV+DMSO, set as 1). **(B)** Representative plaque assay images quantifying infectious VSV particles in supernatants from **(A)**. **(C)** RT-qPCR analysis of viral RNA in HSV-1 (MOI = 0.5), IAV (MOI = 0.1), EMCV (MOI = 0.1)-infected 2fTGH cells as well as MHV-A59 (MOI = 0.1)-infected J774A.1 cells, all treated with either BT (40, 60, or 100 μM) or DMSO (vehicle control) for 12 (h) Data normalized to ACTB and expressed as fold change relative to control (virus+DMSO, set as 1). **(D)** HSV-1 envelope glycoprotein- and MHV-A59 spike protein-mediated cell-cell fusion and syncytium formation in the presence or absence of 60 μM BT for 12 h (Scale bar, 100 μm. The arrow points to fused cells). Data are presented as mean ± SEM, with n = 3. **P < 0.01; ***P < 0.001.

We also determined the anti-VSV activity of BT in HT29, RAW264.7, HeLa, and A549 cells, and the results suggested that BT could broadly inhibit VSV infection across these diverse cell types, as evidenced in **Figures 3A, B**. To extend our analysis to other viruses, we tested BT against DNA virus HSV-1 and RNA viruses such as MHV-A59, EMCV, and IAV. RT-qPCR results showed that BT potently inhibited the infections of these viral strains in a dose-dependent manner, with a concentration of 40 μM reducing viral titers by approximately 50% compared to controls (**Figure 3C**). Moreover, 2fTGH cells infected with HSV-1 exhibited reduced syncytia formation (fewer and smaller in size) following BT treatment, while J774A.1 cells infected with MHV-A59 showed similar suppression of virus-induced cytopathic changes. This implies that BT treatment attenuated membrane fusion events and subsequent cellular damage triggered by viral infection (**Figure 3D**). Collectively, these results indicate that BT effectively suppresses viral infections across a broad spectrum.

### BT inhibits viral replication in an FXa independent manner

**Figure 4:**
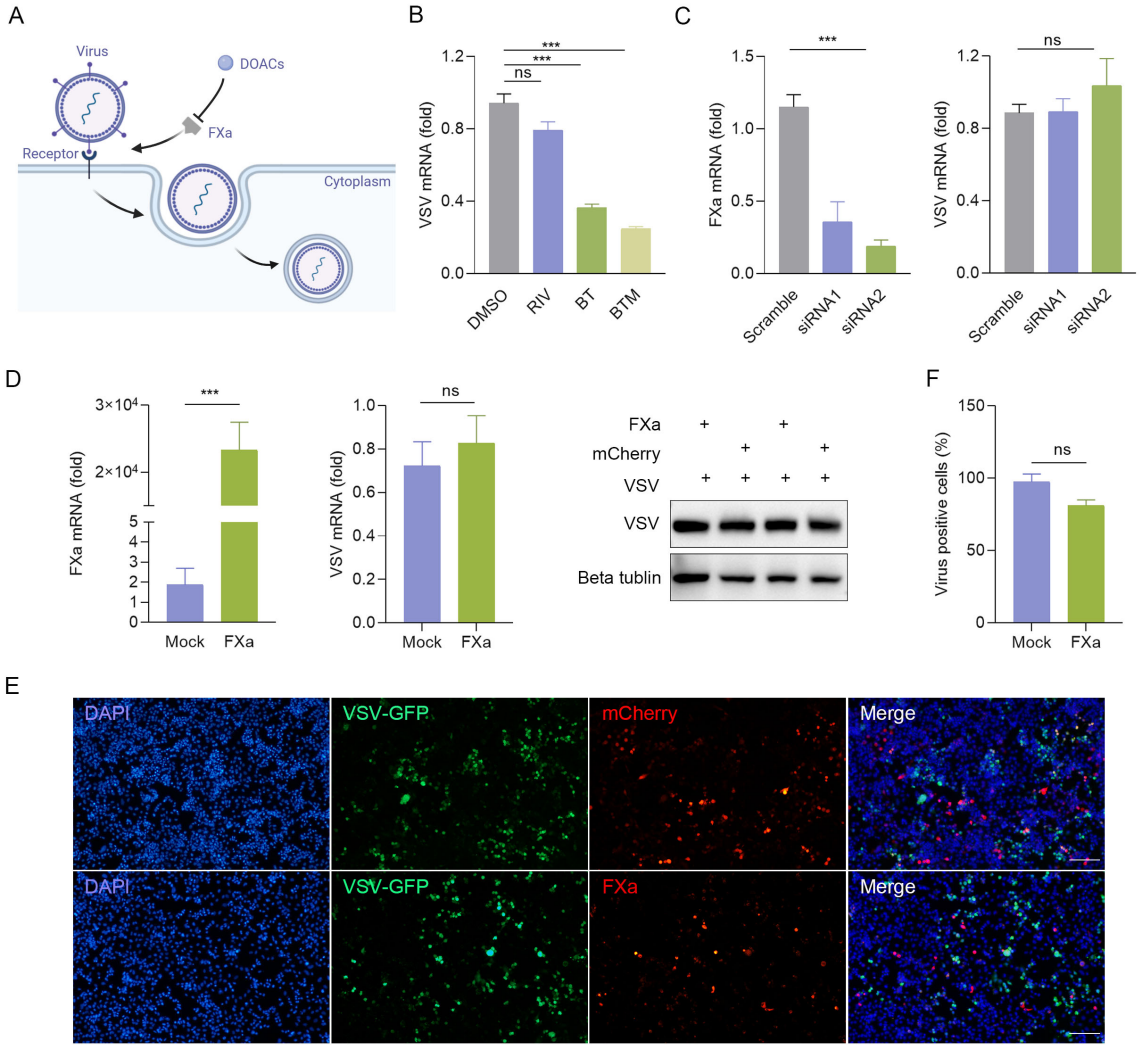
BT arrests viral replication in an FXa independent manner. **(A)** Schematic diagram illustrating the potential antiviral mechanism of BT. **(B)** 2fTGH cells infected with VSV (MOI=0.1) were treated with 50 μM direct oral anticoagulants (DOACs) for 12 h. Antiviral effects quantified by RT-qPCR. Data normalized to ACTB and expressed as fold change relative to DMSO-treated control (set as 1). RIV: Rivaroxaban, BTM: Betrixaban maleate. **(C)** Left: RT-qPCR analysis of FXa mRNA levels in 2fTGH cells transfected with non-targeting control siRNA or FXa siRNA (100 nM). Right: RT-qPCR analysis of VSV-N mRNA levels in 2fTGH cells transfected with non-targeting control siRNA or FXa siRNA (100 nM) followed by VSV infection (MOI = 0.1, 12 h). Data normalized to ACTB and expressed as fold change relative to Scramble-treated (set as 1). **(D)** 2fTGH cells were transfected with 1 μg of FXa-mCherry or an empty plasmid for 48 h, followed by infection with VSV-GFP at an MOI of 0.1 for 12 h. RT-qPCR analysis of FXa mRNA levels and viral replication. Data normalized to ACTB and expressed as fold change relative to empty plasmid-treated (Mock, set as 1). Western blotting analysis of VSV-G protein expression. **(E, F)** HeLa cells were transfected with 1 μg of FXa-mCherry or an empty plasmid for 48 h, followed by infection with VSV-GFP at an MOI of 0.1 for 12 h. Quantification of the percentage of VSV-GFP positive cells in HeLa cells with FXa overexpression via fluorescence microscopy **(E)** and ImageJ **(F)**. Scale bar, 100 μm. The number of VSV-GFP-positive cells in the empty-vector transfection control group (Mock) was normalized to 100% to determine the infection rate. Data are presented as mean ± SEM, with n = 3. ***P < 0.001. ns, not significant.

Previous studies have demonstrated that FXa promotes viral infection by cleaving the SARS-CoV spike protein into its active components, suggesting that FXa inhibitors could potentially serve as effective antiviral agents (Al-Horani, 2020; Frydman et al., 2020; Kastenhuber et al., 2022) (**Figure 4A**). To test this, we compared the inhibitory effects against the virus of a number of direct oral anticoagulants (DOACs), including Rivaroxaban. RT-qPCR detected no change in VSV nucleocapsid protein (VSV-N) mRNA expression in VSV-infected 2fTGH cells with the treatment of Rivaroxaban, whereas BT or Betrixaban maleate (BTM), the maleic acid form of BT, significantly inhibited VSV-N mRNA levels compared to the control group (**Figure 4B**). Similarly, the same result was observed in MHV-A59-infected J774A.1 cells (**Supplementary Figure S4A**), which revealed an unknown mechanism may underlie the antiviral effect of BT. Furthermore, we investigated whether altering the expression of FXa, the only known target protein of BT, had an impact on the antiviral activity. To this end, FXa was either overexpressed or knocked down in 2fTGH via transient transfection with an FXa-mCherry plasmid (**Supplementary Figure S4B**) or siRNA, and the cells were then infected with VSV. The results showed that the change of the expression level of FXa did not significantly impact BT’s antiviral effects (**Figures 4C–F**). Taken together, these data demonstrate that the antiviral activity of BT is independent of FXa protein function.

### BT activates the IFN-I pathway and initiates antiviral responses *in vitro*

**Figure 5:**
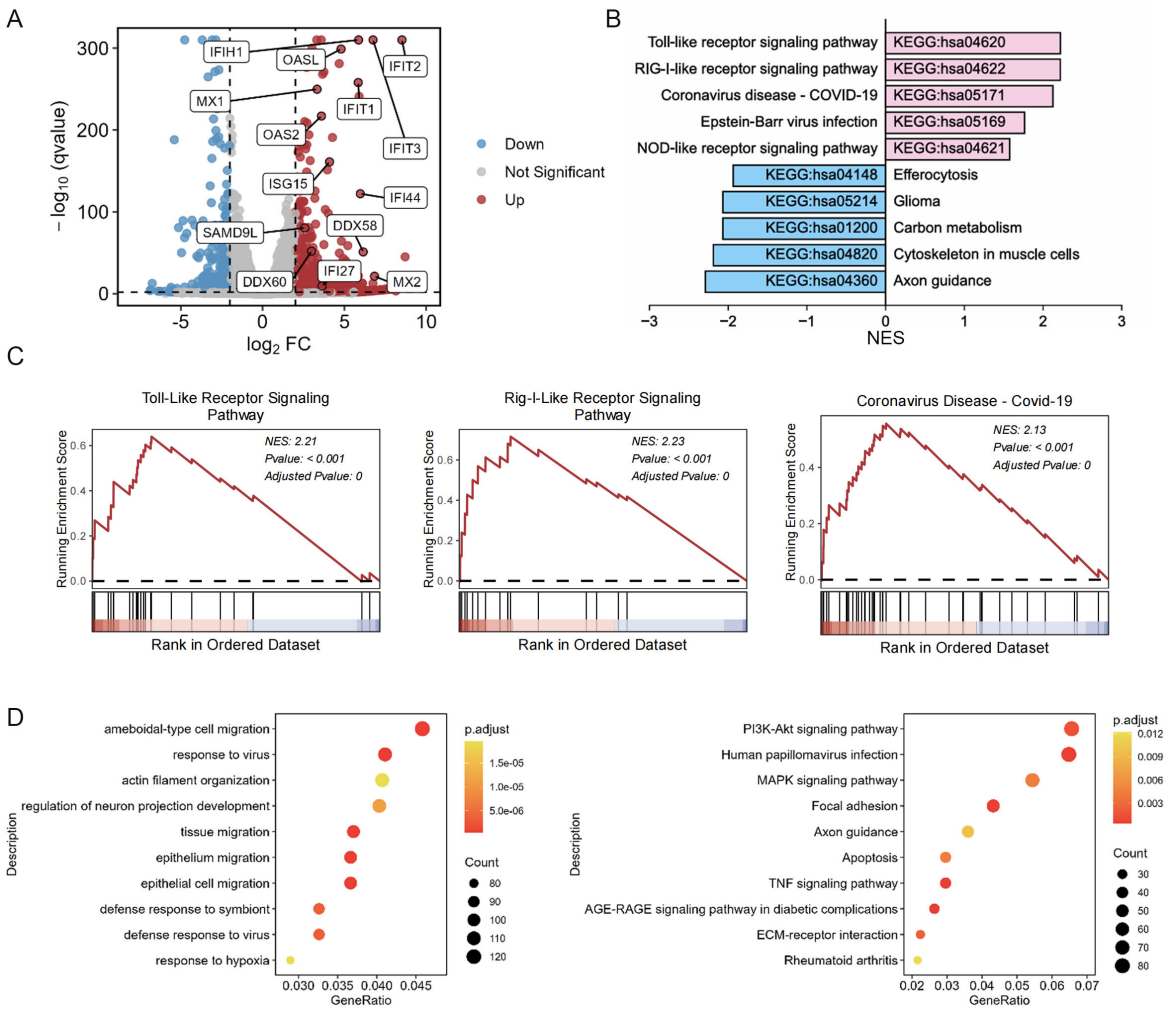
BT induces upregulation of ISGs associated with antiviral protection. 2fTGH cells were exposed to 100 µM BT or DMSO for 12 h. RNA sequencing was then performed to analyze the resulting transcriptomic changes. **(A)** Volcano plots of differentially expressed genes (DEGs) in 2fTGH cells treated with BT compared to DMSO-treated control cells. ISGs are highlighted in blue (downregulated) and red (upregulated). **(B)** The enrichment of KEGG of BT-induced up- and down-regulated genes was presented. **(C)** GSEA was performed on BT-induced gene expression profiles. The results include normalized enrichment scores (NES) and nominal p-values. **(D)** The bubble plot of enrichment analysis of DEGs using over-representation analysis (ORA) method. Left: GO-Biological Process (GO-BP) enrichment analysis. Right: KEGG pathway enrichment analysis.

To elucidate the underlying mechanism of the antiviral efficacy of BT, we performed RNA-seq to analyze global transcriptome changes in 2fTGH cells after BT treatment. Compared with the control group, the upregulated differentially expressed genes (DEGs) included several ISGs associated with antiviral immune responses, including myxovirus resistance proteins (Mx), IFN-stimulated protein of 15 kDa (ISG15), interferon-induced tetrapeptide repeat family proteins (IFIT), and 2’-5’-oligoadenylate synthetase 2 (OAS2) (**Figure 5A**). KEGG pathway enrichment analysis and GSEA revealed that BT upregulated antiviral immune-related pathways, such as the Toll-like receptor signaling pathway and the RIG-I-like receptor signaling pathway (**Figures 5B, C**). Bubble plots from enrichment analysis of DEGs using KEGG and GO-BP databases via over-representation analysis (ORA) highlighted antiviral-related pathways (**Figure 5D**). In addition, RT-qPCR analysis confirmed the RNA-seq results, demonstrating that some annotated ISGs were markedly upregulated following BT treatment, with a 10- to 200-fold increase observed compared to controls (**Figure 1E**). Together, these findings imply that BT positively modulates the antiviral immune response. Furthermore, transcriptomic profiling revealed multiple significantly downregulated pathways following BT treatment, including metabolism (carbon metabolism, glycerolipid metabolism, Biosynthesis of amino acids), cellular signaling and structural integrity (axon guidance, gap junction formation, cytoskeletal organization in muscle cells) and disease-related pathways (glioma, renal cell carcinoma, pancreatic secretion) (**Figure 5B**; **Supplementary Table S2**). These results indicate that BT treatment triggers a comprehensive regulatory response, involving not only immune activation but also suppression of pathways related to cell metabolism, tumor-associated networks, and specific signaling networks.

**Figure 6:**
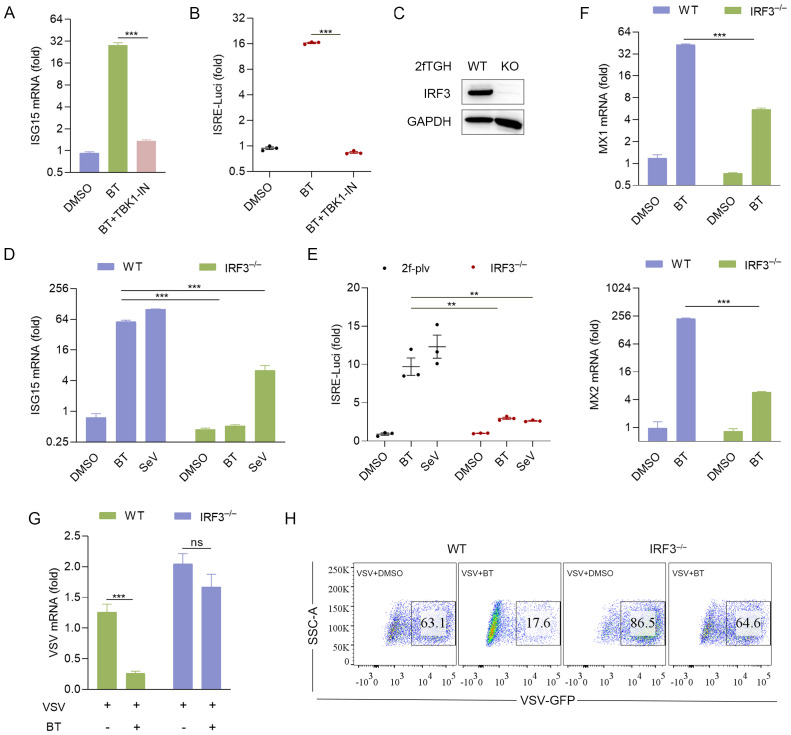
BT elicits antiviral immune response via TBK1/IRF3 signaling axis. **(A)** RT-qPCR analysis of ISG15 mRNA expression in 2fTGH cells pretreated with TBK1 inhibitor for 6 h, followed by treatment with BT (100 μM, 12 h). Data normalized to ACTB and expressed as fold change relative to DMSO-treated control (set as 1). **(B)** Luciferase activity in 2fTGH cells treated as described in **(A)**. Relative luciferase activity was expressed as fold change relative to the control (DMSO, set as 1.0). **(C)** IRF3 protein levels in IRF3 knockout (IRF3^–/–^) 2fTGH cells and WT cells. **(D, E)** The Luciferase reporter activity and gene expression of ISG15 in WT and IRF3^–/–^ 2fTGH cells after 16 h of BT (60 μM) or SeV (MOI = 0.1) treatment. Data normalized to ACTB and expressed as fold change relative to DMSO-treated control (set as 1). Relative luciferase activity was expressed as fold change relative to the control (DMSO, set as 1.0). **(F)** RT-qPCR analysis of Mx mRNA levels in WT and IRF3^–/–^ 2fTGH cells following treatment with BT (60 μM) for 16 h. Data normalized to ACTB and expressed as fold change relative to DMSO-treated control (set as 1). **(G, H)** RT-qPCR **(G)** and flow cytometry analysis **(H)** evaluating virus (VSV or VSV-GFP, MOI = 0.1) infection in WT and IRF3-depleted 2fTGH cells treated with BT (60 μM) for 12 h. Data normalized to ACTB and expressed as fold change relative to VSV-treated control (set as 1). Data are presented as mean ± SEM, with n = 3. **P < 0.01; ***P < 0.001. ns, not significant.

To further investigate the intracellular activation of the IFN-I pathway by BT *in vitro*, a TBK1 inhibitor, GSK8612 (TBK1-IN), was added to 2fTGH cells treated with BT. Additionally, IRF3 knockout (IRF3^–/–^) 2fTGH cell line and Irf3^–/–^ BMDMs were treated with BT. Interestingly, the luciferase activity and ISG15 mRNA transcription levels in 2fTGH cells were notably suppressed after TBK1-IN treatment (**Figures 6A, B**). Consistently, a significant reduction in luciferase and ISG mRNA levels was observed in both IRF3^–/–^ 2fTGH cells and Irf3^–/–^ BMDMs following BT treatment compared to wild-type (WT) cells (**Figures 1G**, **6C–E**), establishing that BT modulates the innate immune signaling pathway through the TBK1/IRF3 axis.

We subsequently hypothesized that the upregulation of BT-induced ISGs correlates with its antiviral protective effects. To test this hypothesis, we examined the expression of antiviral-related genes in IRF3^–/–^ 2fTGH cells. RT-qPCR analysis showed that the transcript levels of Mx genes, including Mx1 and Mx2, increased by at least tenfold upon BT treatment, whereas this upregulation was absent in IRF3^–/–^ cells (**Figure 6F**). Furthermore, BT displayed significantly weaker inhibition against VSV or VSV-GFP in IRF3^–/–^ cells compared to WT cells (**Figures 6G, H**). These findings indicate that BT’s antiviral efficacy is markedly reduced in the absence of IRF3, highlighting the critical role of the TBK1/IRF3 axis in BT-mediated antiviral activity.

### BT protects mice against VSV infection *in vivo*

**Figure 7:**
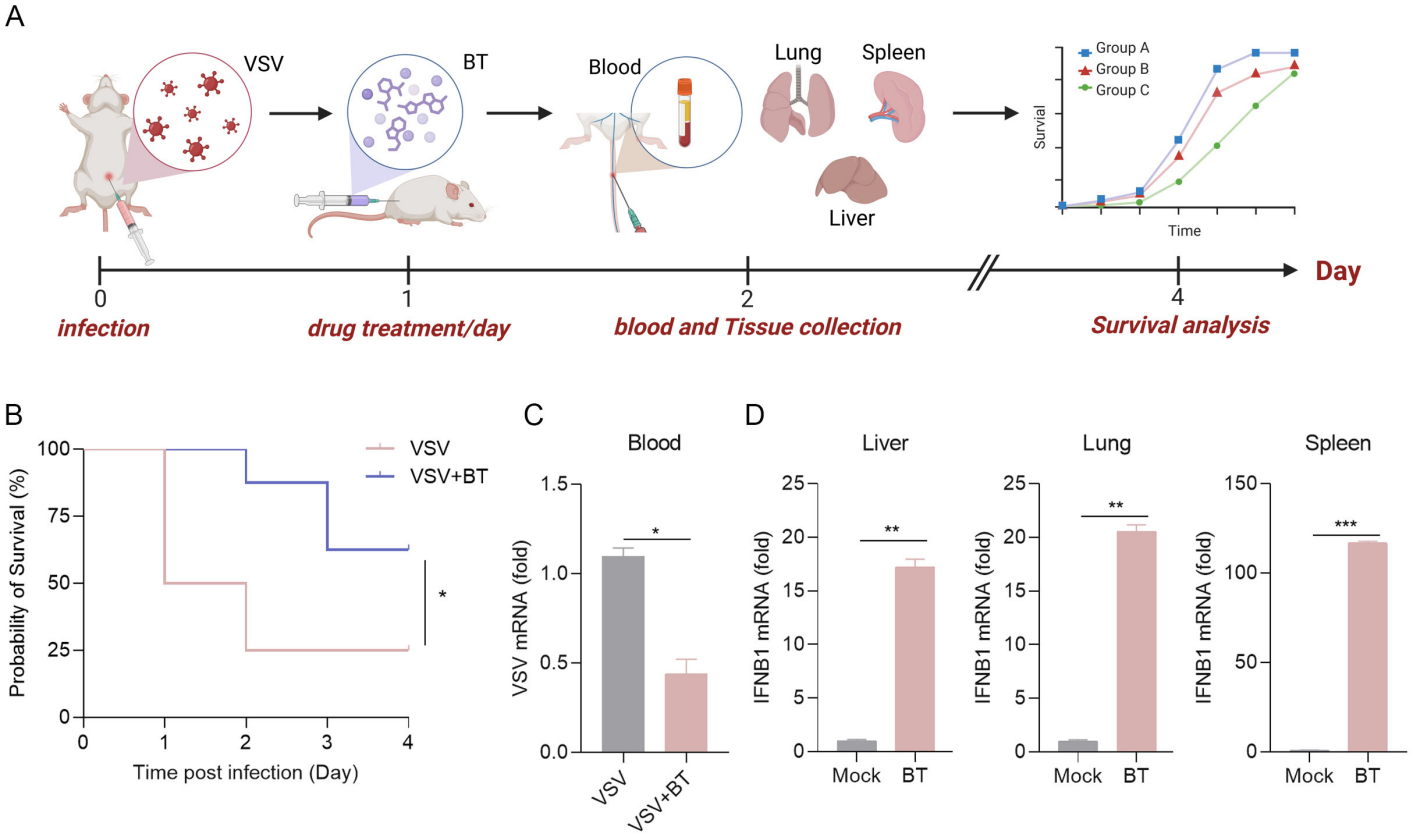
The antiviral protective effects of BT treatment *in vivo*. **(A)** Schematic representation of a mouse model of VSV infection treated with BT *in vivo*. C57BL/6 mice were infected intraperitoneally (i.p.) with VSV (1 × 10^8^ PFU per mouse), followed by daily i.p. injections of BT (10 mg/kg/day) or PBS (vehicle control) for three consecutive days. **(B)** Survival curves of VSV-infected mice treated with BT or PBS (n = 8 per group). Survival comparisons were performed using the log-rank (Mantel-Cox) test. **(C)** RT-qPCR analysis of the VSV-N mRNA levels in the blood tissues of VSV-infected mice treated with BT or not (n = 3 per group). Data normalized to ACTB and expressed as fold change relative to PBS-treated control (VSV alone, set as 1). **(D)** IFNB1 mRNA levels in the liver, lung, and spleen tissues harvested from C57BL/6J mice 24 h after a single i.p. injection of BT (10 mg/kg) or PBS were quantified by RT-qPCR (n = 3 per group). Data normalized to ACTB and expressed as fold change relative to PBS-treated control (Mock, set as 1). Data are presented as mean ± SEM, with n = 3. *P < 0.05; **P < 0.01; ***P < 0.001.

To further explore the physiological antiviral function of BT *in vivo*, WT C57BL/6 mice were intraperitoneally infected with VSV and subsequently treated daily with either PBS or BT (10 mg/kg/day) for three consecutive days (**Figure 7A**). Remarkably, the survival rate of WT mice in the BT treatment group was significantly higher compared to that of the control group (**Figure 7B**). To determine whether BT inhibited VSV replication in tissues, the levels of viral RNA in the blood were measured using RT-qPCR. The results showed that the levels of VSV-N mRNA in the blood of mice receiving BT treatment were reduced compared to the control group (**Figure 7C**). Interestingly, RT-qPCR analysis demonstrated a rapid activation of IFNB1 mRNA expression in the tissues of mice following a single dose of BT treatment as compared to the control group, indicating BT’s ability to activate innate immune pathways *in vivo* (**Figure 7D**). In conclusion, our findings demonstrate that BT is protective against VSV infection *in vivo*.

## Discussion

Recently, changes in the natural environment and the expansion of human activities have contributed to frequent infectious disease occurrences, including seasonal influenza, monkeypox, dengue fever, and Zika fever (Talemi et al., 2021; Adler et al., 2022). Emerging viral threats such as Ebola hemorrhagic fever and Nipah virus disease further challenge global public health (Weaver et al., 2018; Roy et al., 2023). These viral infections are often associated with high morbidity and mortality rates, with some having the potential to trigger global pandemics (Wu et al., 2020). The emergence of SARS-CoV-2 underscores the urgent need for effective antiviral therapeutics. This novel enveloped virus, belonging to the Coronaviridae family, which includes other pathogenic members such as SARS-CoV and Middle East respiratory syndrome coronavirus (MERS-CoV), causes a wide spectrum of clinical manifestations, ranging from mild respiratory symptoms to severe pneumonia, acute respiratory distress syndrome, and multi-organ failure. Disease severity is often exacerbated by hypercoagulability, characterized by elevated D-dimer levels and thrombocytopenia, both independent predictors of poor outcomes. The underlying coagulopathy involves viral and host factors, including coagulation cascade activation and inflammatory responses (Elahi, 2022). Consequently, antiviral therapies targeting coagulation processes may offer dual benefits by modulating both inflammation and coagulation, thus enhancing treatment outcomes in viral infections.

As research progresses, direct factor Xa (FXa) inhibitors, commonly referred to as “xaban” drugs, have garnered attention not only for their significant anticoagulant properties but also for their potential antiviral effects. FXa-specific direct oral anticoagulants (DOACs) demonstrate antiviral and anti-inflammatory activities in mouse models of disease (Sutherland et al., 2019). When administered early during viral infections, these drugs exhibit combined antiviral, anti-inflammatory, and anticoagulant effects. In contrast, during late-stage infections, they primarily alleviate thrombotic and inflammatory complications (Frydman et al., 2020). FXa, as a major target of DOAC, exhibits antiviral effects against various viruses in previous studies. For instance, FXa is essential for hepatitis E virus replication in Huh7-S10-3 cells by participating in the processing of the ORF1 polyprotein; therefore, direct FXa inhibitors may disrupt hepatitis E virus replication (Kanade et al., 2018). In HUVEC cells, FXa utilizes herpes simplex virus (HSV)-associated tissue factor (TF) to enhance viral infection through cellular protease-activated receptor (PAR) (Sutherland et al., 2004). In SARS-CoV-infected Vero cells, FXa specifically cleaves and activates the viral spike protein (S protein), facilitating virion entry, while FXa inhibitors block SARS-CoV plaque formation in a concentration-dependent manner. Therefore, direct FXa inhibitors can effectively prevent SARS-CoV entry into host cells (Du et al., 2007).

Here, among the various candidates, Betrixaban (BT), an oral anticoagulant, has garnered significant interest due to its notable antiviral properties and immunoregulatory activity, as identified in a luciferase-based screening assay. Although BT has previously been proposed as a new potential inhibitor of SARS-CoV-2 (Al-Horani, 2020; Pryzdial et al., 2020), its precise antiviral mechanisms and immune-modulatory effects remain largely unexplored. Our study showed a substantial inhibitory property of BT in restricting the replication of DNA and RNA viruses (HSV-1, IAV, MHV, EMCV, and VSV) *in vitro* in a concentration-dependent manner. Moreover, *in vivo* experiments using VSV-infected mice demonstrated that BT not only improves survival rates following viral infection but also inhibits the replication of VSV, underscoring its therapeutic potential in viral diseases.

Interestingly, previous studies predicted that BT inhibits SARS-CoV-2 infection by targeting host proteases such as FXa or TMPRSS. However, our findings provide new insight into BT’s mechanism of action, revealing that its antiviral effects are primarily mediated through innate immune activation rather than direct inhibition of viral entry. Our data show that BT promotes IFN-β production and induces the expression of multiple interferon-stimulated genes (ISGs), thereby enhancing the host’s antiviral defense. The antiviral efficacy of BT was significantly reduced in IRF3^–/–^ cells, where the induction of key ISGs, such as Mx1 and Mx2, was abolished. Collectively, these results confirm the essential role of innate immune activation in BT-mediated antiviral protection.

To gain deeper insights into the cellular mechanisms of immune activation, we analyzed BT-induced transcriptomic landscape via RNA-seq. Our results highlighted significantly upregulated genes involved in antiviral and innate immune responses. Concurrently, we observed downregulation of genes related to metabolic pathways (e.g., glycerolipid metabolism, phosphatidylinositol signaling), cellular signaling cascades (e.g., Rap1 and Hippo signaling), and structural organization systems (gap junctions, cytoskeletal organization, axon guidance), which indicates a comprehensive reprogramming of cellular physiology that may contribute to its therapeutic effects. Additionally, pathways associated with tumor progression and drug resistance, such as EGFR tyrosine kinase inhibitor resistance, glioma, renal cell carcinoma, and efferocytosis, were also notably suppressed. These findings raise intriguing possibilities for broader therapeutic implications of BT beyond antiviral effects, potentially extending to cancer biology and modulation of drug resistance mechanisms. Further targeted studies are warranted to explore the precise biological significance and clinical relevance of these transcriptional changes.

This study advances the understanding of small-molecule modulators of innate immunity. On one hand, BT represents a promising immunomodulatory compound with broad-spectrum antiviral potential, which is particularly valuable in the context of emerging infectious disease outbreaks. On the other hand, its efficacy *in vivo* further supports the need for clinical exploration of BT or its derivatives as antiviral agents. Nevertheless, excessive type I interferon (IFN-I) responses and ISGs induction may lead to deleterious inflammatory consequences, including autoimmune reactions or inflammatory tissue damage (Weiss et al., 2017; Yang et al., 2021; Fernandez-Ruiz and Niewold, 2022). Previous studies have reported that sustained IFN-I signaling can contribute to chronic inflammation, autoimmunity, and impaired tissue repair, raising potential safety concerns for therapies that strongly activate innate immunity (Rodero and Crow, 2016; Wu, 2016; Muskardin and Niewold, 2018; Barrat et al., 2019). Therefore, further clinical studies are needed to determine the balance between therapeutic benefits and possible adverse effects associated with BT-induced immune activation.

Furthermore, our current study primarily investigates acute viral infection models; the therapeutic efficacy of BT in chronic viral infections remains unexplored. Chronic viral infections, such as those caused by Epstein-Barr Virus (EBV), hepatitis B virus (HBV), and hepatitis C virus (HCV), are characterized by persistent immune activation and dysregulated antiviral responses, which differ from those of acute infections (Saeed et al., 2014; Shin et al., 2016). Thus, investigating BT’s antiviral effects and immunomodulatory properties in chronic infection models will provide critical insights into its broader therapeutic potential and limitations. Future research should evaluate BT in established animal models of chronic infection, assessing not only antiviral efficacy but also potential long-term immunological complications to ensure the safe and effective clinical translation of BT.

It is also noteworthy that BT induced IFN-β expression at concentrations well above those needed for its anticoagulant activity. This finding suggests that BT may act through mechanisms independent of FXa, its classical anticoagulant target. In fact, we found that the same concentration of Rivaroxaban failed to induce IFN-β expression (**Supplementary Figure S1F**). BT may directly or indirectly activate pattern recognition receptors (PRRs), such as the RIG-I-like receptor, Toll-like, or the cGAS-STING pathway, thus triggering the production of IFN-I. The biological significance of this potential mechanism may relate to immunomodulatory activities of BT at elevated concentrations, independent of its anticoagulant function. However, more evidence is required to specify BT’s preferential activation pathways leading to IFN-I production in the future.

In conclusion, our work demonstrates that BT acts as a novel immunomodulator, enhancing antiviral immunity by activating the IFN-I response. These findings highlight BT’s function as a broad-spectrum antiviral agent, making it a promising candidate for future drug development against various viral infections.

## Data Availability

The original contributions presented in the study are included in the article/**Supplementary Material**, further inquiries can be directed to the corresponding author/s.

## Supplementary material

Supplementary Figure 1
**(A–D)** Cell viability assessed by CCK-8 assay after BT treatment (0-200 μM) for 12 h **(A)**, 24 h **(B)**, 36 h **(C)**, or 48 h **(D)**. Mock group (untreated) served as negative control (set as 100% viability). **(E)** Luciferase activity in 2fTGH cells with BT treatment at different concentrations (10, 30, 60, or 100 μM) for 12 h. **(F)** Luciferase activity in 2fTGH cells after 12-h treatment with 60 μM BT, Rivaroxaban, or DMSO. Data expressed as fold change vs. DMSO control. Data are presented as mean ± SEM, with n = 3. *P < 0.05; **P < 0.01; ***P < 0.001. ns, not significant.

Supplementary Figure 2
**(A)** Cytopathic effects in 2fTGH cells infected with VSV (MOI = 0.1, 24 h) and treated with BT (60 μM) or DMSO control. Scale bar, 100 μm. **(B)** Visualization of the plaque reduction assay. Supernatants from VSV-infected cells treated with BT (0-100 μM) serially diluted, adsorbed onto fresh 2fTGH cells (1 h), overlaid with 0.5% carboxymethyl cellulose, and incubated for 48 h before plaque staining. **(C)** Flow cytometry of VSV-GFP-positive 2fTGH cells infected with VSV-GFP (MOI = 0.1, 24 h) and treated with BT (60 μM) or IFN-β (1000 U/mL, positive control). **(D)** Representative green fluorescence images of 2fTGH cells infected with VSV-GFP (MOI = 0.1, 12 h) with BT (60 μM) or IFN-β (1000 U/mL); nuclei counterstained with DAPI (blue); scale bar: 100 μm. Data are presented as mean ± SEM, with n = 3. *P < 0.05; **P < 0.01; ***P < 0.001. ns, not significant.

Supplementary Figure 3
**(A–D)** Cell viability of A549 **(A)**, HeLa **(B)**, HT29 **(C)**, and Raw264.7 **(D)** cells treated with different BT concentrations (0-200 μM) for 24 h was assessed by CCK-8 assay. Untreated cells (Mock) cultured in parallel served as negative controls (defined as 100% viability).

Supplementary Figure 4
**(A)** J774A.1 cells were infected with MHV-A59 (MOI = 0.1), and treated with 50 μM DOACs for 12 h. RT-qPCR quantified viral RNA levels. Gene expression data were normalized to ACTB. Fold changes relative to DMSO-treated control samples were calculated using the 2^-ΔΔCT method. **(B)** 293T cells transfected with 1 μg FXa-mCherry or empty plasmid for 24 h. FXa-mCherry fusion protein expression and cellular localization were presented by Western blotting and fluorescence microscopy. Antibody, mCherry. Scale bar, 100 μm. Data are presented as mean ± SEM, with n = 3. *P < 0.05; **P < 0.01; ***P < 0.001. ns, not significant.
