# Supplementary figures and images for: Betrixaban is a broad anti-virus inhibitor by activating innate immunity
Source: Front Cell Infect Microbiol. 2025 Aug 21;15:1603530. doi: 10.3389/fcimb.2025.1603530 (PMC12408630; doi:10.3389/fcimb.2025.1603530)

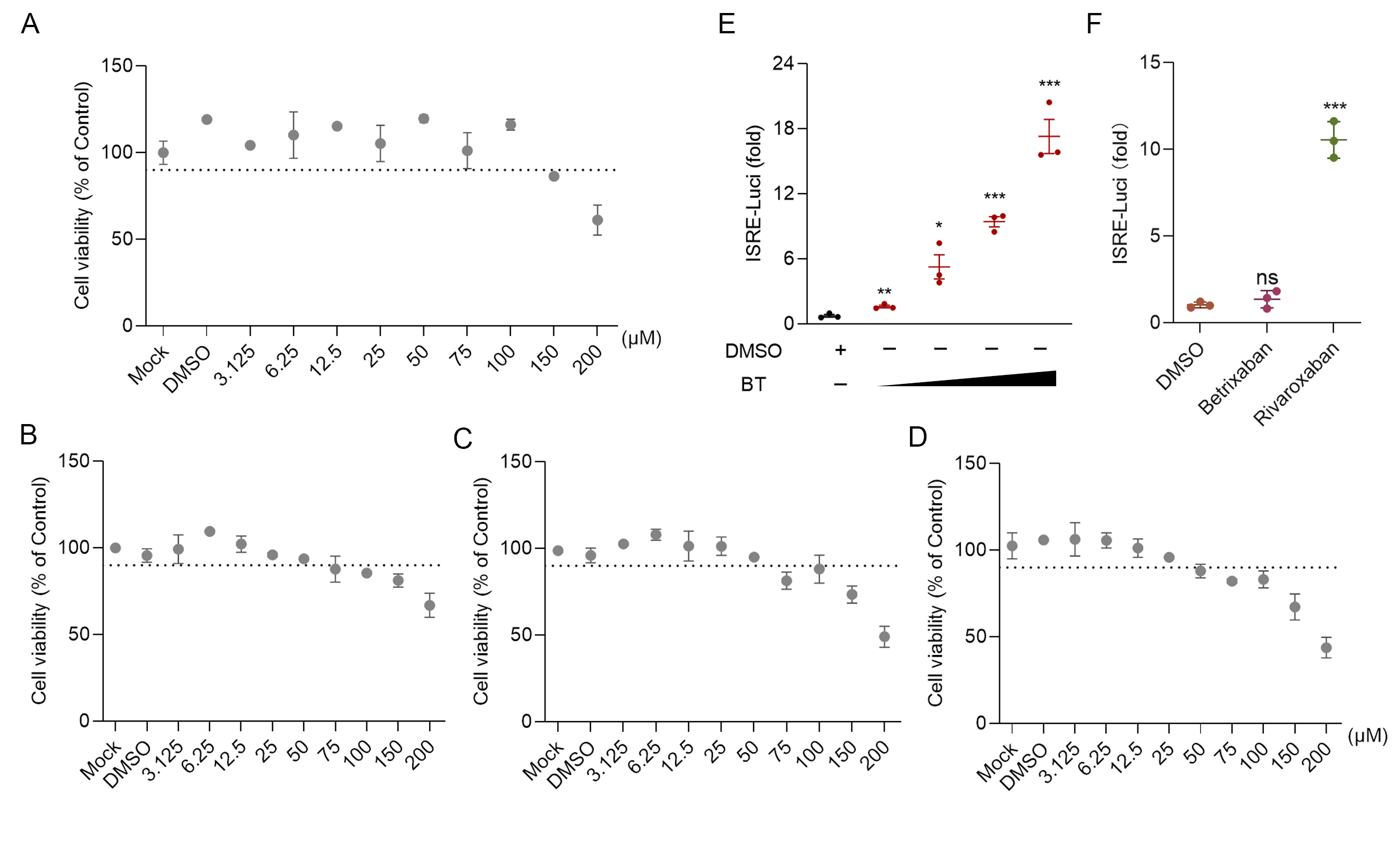

Supplement: Supplementary Figure 1 — (A–D) Cell viability assessed by CCK-8 assay after BT treatment (0-200 μM) for 12 h (A), 24 h (B), 36 h (C), or 48 h (D). Mock group (untreated) served as negative control (set as 100% viability). (E) Luciferase activity in 2fTGH cells with BT treatment at different concentrations (10, 30, 60, or 100 μM) for 12 h. (F) Luciferase activity in 2fTGH cells after 12-h treatment with 60 μM BT, Rivaroxaban, or DMSO. Data expressed as fold change vs. DMSO control. Data are presented as mean ± SEM, with n = 3. *P < 0.05; **P < 0.01; ***P < 0.001. ns, not significant. [file Image1.tif]

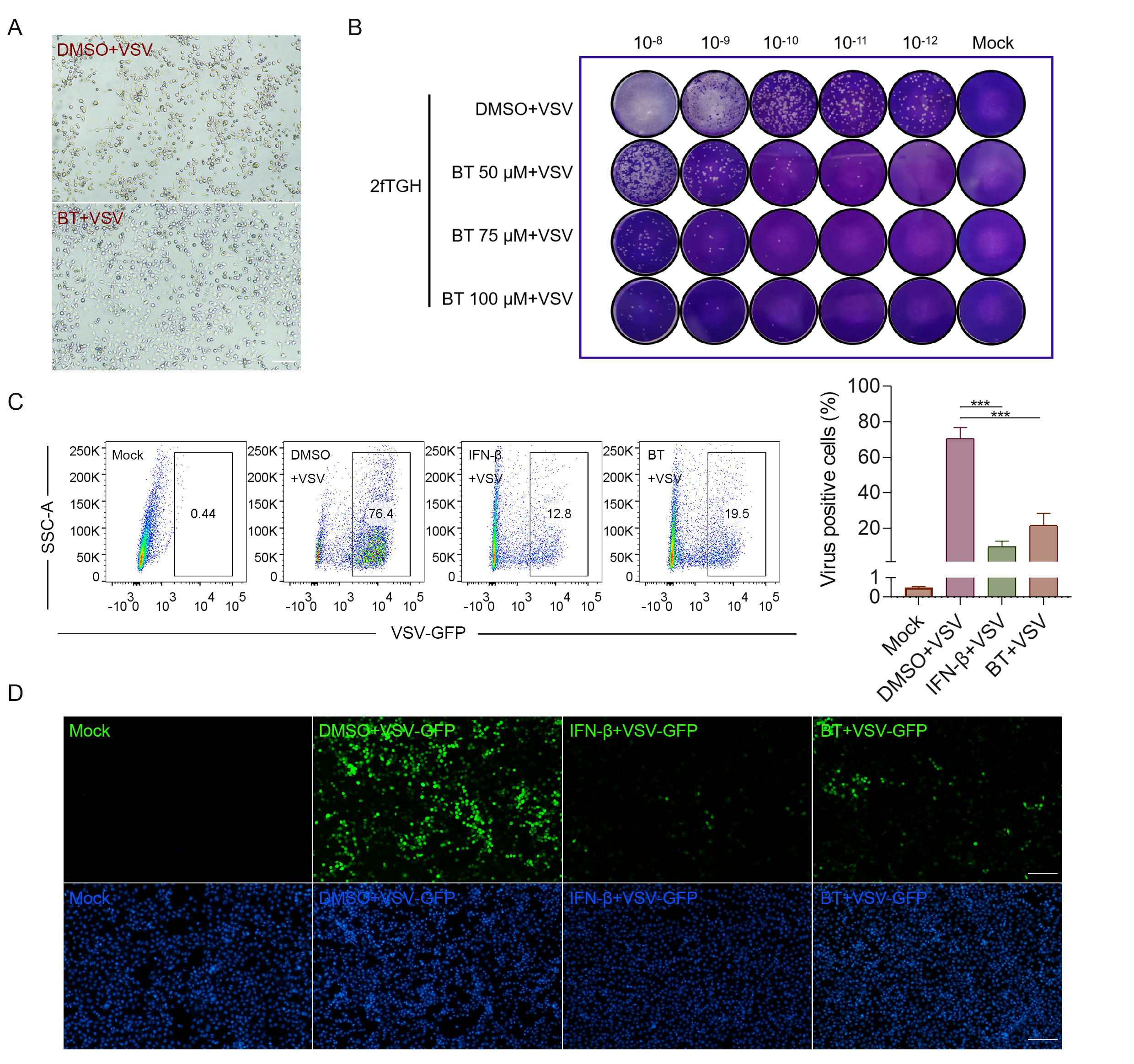

Supplement: Supplementary Figure 2 — (A) Cytopathic effects in 2fTGH cells infected with VSV (MOI = 0.1, 24 h) and treated with BT (60 μM) or DMSO control. Scale bar, 100 μm. (B) Visualization of the plaque reduction assay. Supernatants from VSV-infected cells treated with BT (0-100 μM) serially diluted, adsorbed onto fresh 2fTGH cells (1 h), overlaid with 0.5% carboxymethyl cellulose, and incubated for 48 h before plaque staining. (C) Flow cytometry of VSV-GFP-positive 2fTGH cells infected with VSV-GFP (MOI = 0.1, 24 h) and treated with BT (60 μM) or IFN-β (1000 U/mL, positive control). (D) Representative green fluorescence images of 2fTGH cells infected with VSV-GFP (MOI = 0.1, 12 h) with BT (60 μM) or IFN-β (1000 U/mL); nuclei counterstained with DAPI (blue); scale bar: 100 μm. Data are presented as mean ± SEM, with n = 3. *P < 0.05; **P < 0.01; ***P < 0.001. ns, not significant. [file Image2.tif]

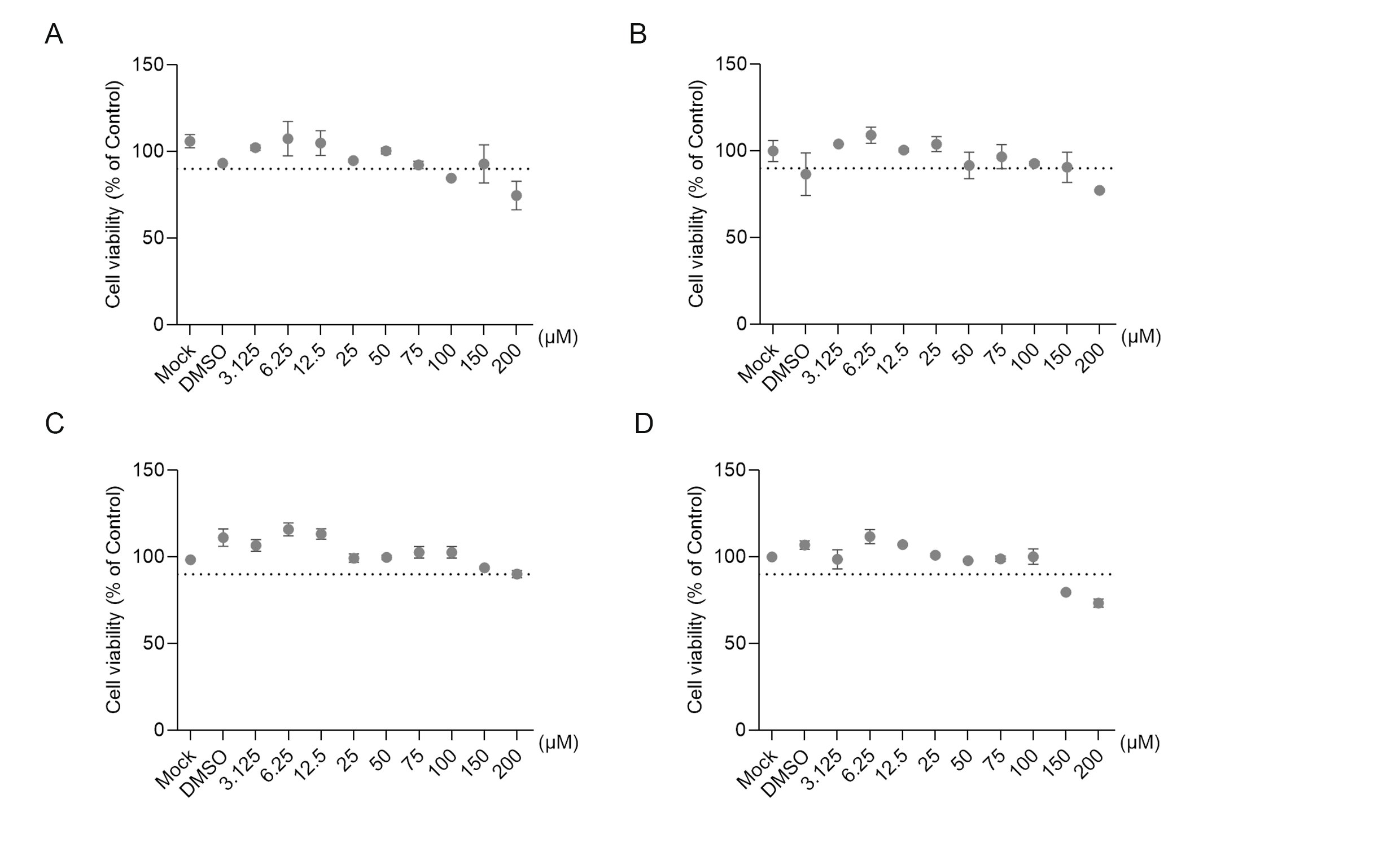

Supplement: Supplementary Figure 3 — (A–D) Cell viability of A549 (A), HeLa (B), HT29 (C), and Raw264.7 (D) cells treated with different BT concentrations (0-200 μM) for 24 h was assessed by CCK-8 assay. Untreated cells (Mock) cultured in parallel served as negative controls (defined as 100% viability). [file Image3.tif]

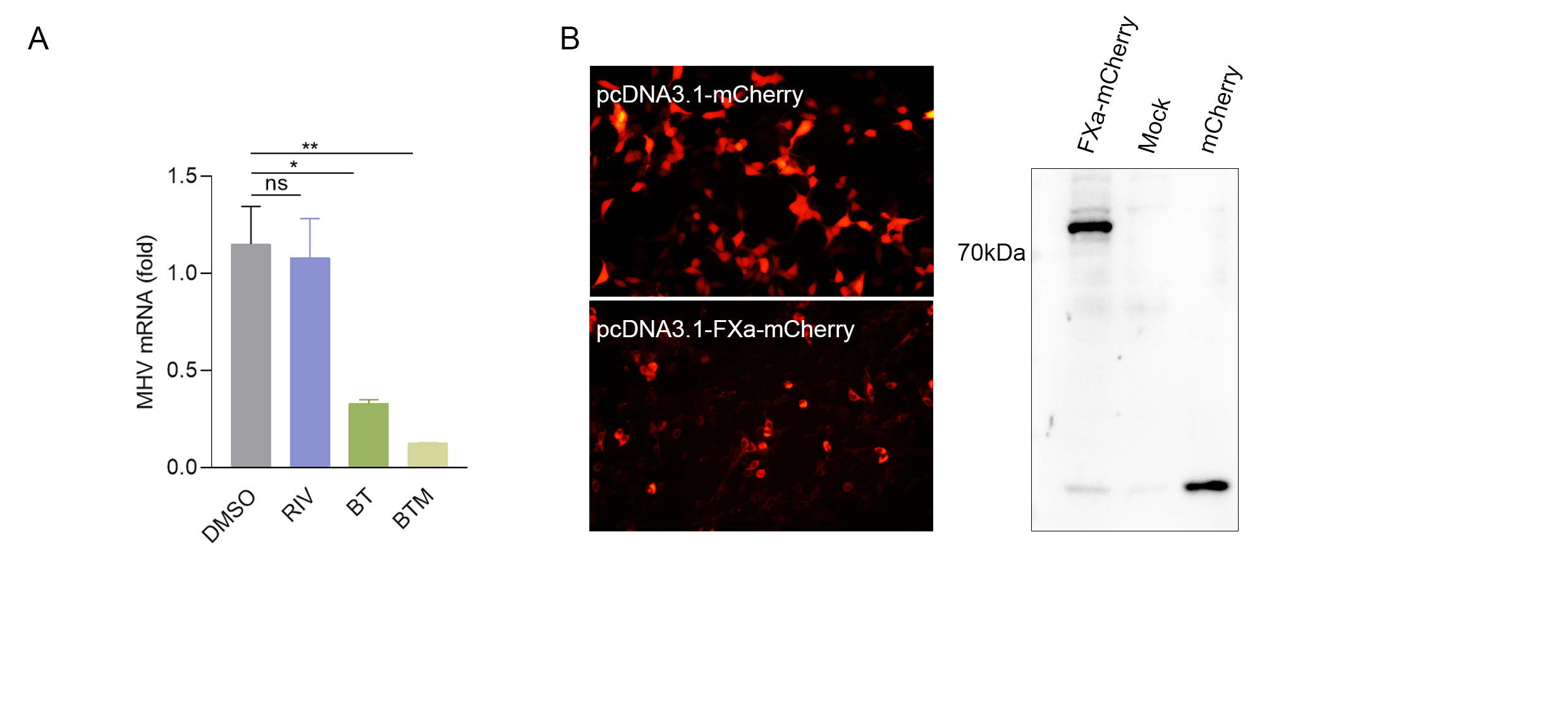

Supplement: Supplementary Figure 4 — (A) J774A.1 cells were infected with MHV-A59 (MOI = 0.1), and treated with 50 μM DOACs for 12 h. RT-qPCR quantified viral RNA levels. Gene expression data were normalized to ACTB. Fold changes relative to DMSO-treated control samples were calculated using the 2^-ΔΔCT method. (B) 293T cells transfected with 1 μg FXa-mCherry or empty plasmid for 24 h. FXa-mCherry fusion protein expression and cellular localization were presented by Western blotting and fluorescence microscopy. Antibody, mCherry. Scale bar, 100 μm. Data are presented as mean ± SEM, with n = 3. *P < 0.05; **P < 0.01; ***P < 0.001. ns, not significant. [file Image4.tif]
